# Iron-Induced Hepatocarcinogenesis—Preventive Effects of Nutrients

**DOI:** 10.3389/fonc.2022.940552

**Published:** 2022-06-27

**Authors:** Hiroyuki Tsuchiya

**Affiliations:** Division of Molecular and Genetic Medicine, Graduate School of Medicine, Tottori University, Yonago, Japan

**Keywords:** hepatic iron overload, nutrients, nutritional prevention, hepatocellular carcinoma, chronic liver diseases

## Abstract

The liver is a primary organ that stores body iron, and plays a central role in the regulation of iron homeostasis. Hepatic iron overload (HIO) is a prevalent feature among patients with chronic liver diseases (CLDs), including alcoholic/nonalcoholic liver diseases and hepatitis C. HIO is suggested to promote the progression toward hepatocellular carcinoma because of the pro-oxidant nature of iron. Iron metabolism is tightly regulated by various factors, such as hepcidin and ferroportin, in healthy individuals to protect the liver from such deteriorative effects. However, their intrinsic expressions or functions are frequently compromised in patients with HIO. Thus, various nutrients have been reported to regulate hepatic iron metabolism and protect the liver from iron-induced damage. These nutrients are beneficial in HIO-associated CLD treatment and eventually prevent iron-mediated hepatocarcinogenesis. This mini-review aimed to discuss the mechanisms and hepatocarcinogenic risk of HIO in patients with CLDs. Moreover, nutrients that hold the potential to prevent iron-induced hepatocarcinogenesis are summarized.

## Introduction

Iron is an essential micronutrient that is utilized as a co-factor for various proteins, including heme and Fe-S proteins (1, 2). However, iron facilitates hydroxyl radical production *via* a well-established mechanism, the Fenton reaction (3). Hydroxyl radical is one of the most potent reactive oxygen species, which harshly damages cellular components, including nucleic acids, proteins, and lipids, leading to the collapse of cellular homeostasis. Moreover, excessive cellular iron causes ferroptosis, a nonapoptotic programmed cell death, which is recently suggested to be involved in the development of a broad range of diseases, including chronic liver diseases (CLDs) (4). Thus, iron metabolism is precisely controlled by various factors, such as hepcidin and ferroportin (1, 2, 4).

Body iron is mainly stored in the liver; thus, compromised function and expression of these iron metabolism-related factors readily cause hepatic iron overload (HIO). Hereditary hemochromatosis, which leads to massive iron accumulation not only in liver, but also in many other organs, such as heart and pancreas, etc., is caused by genetic defects in the iron metabolism-related factors that frequently result in diabetes mellitus, cardiomyopathy, and liver cancer (5). Moreover, nonhereditary, secondary HIO is prevalent among patients with CLDs, such as chronic hepatitis C, alcoholic liver disease, and nonalcoholic fatty liver disease (NAFLD), all of which are important etiologies of hepatocellular carcinoma (HCC) (6). As mentioned above, excessive iron severely impairs normal tissue functions by aggravating oxidative stress; thus, HIO is suggested to promote the development and progression of these CLDs and even predispose them to HCC. Contrastingly, several lines of evidence indicate that the correction of dysregulated iron metabolism significantly improves liver functions and ameliorates pathologies related to CLDs associated with HIO. Therefore, HCC is reasonably expected to arise from HIO-associated CLDs, which can be prevented by interventions that target iron metabolism.

The present mini-review briefly described the current knowledge on HIO associated with CLDs, focusing on mechanisms and hepatocarcinogenesis. Moreover, nutritional interventions with protective effects against HIO by correcting iron dysmetabolism are concisely summarized.

## HIO in CLDs

HIO is attributable to both genetic and nongenetic causes. Hemochromatosis results from genetic defects of iron-metabolism-related genes, including *HFE*, *HAMP* (encoding hepcidin), *HJV* (hemojuvelin), *TFR2* (transferrin receptor 2*)*, and *SLC40A1* (ferroportin*)* genes (5), whose functions are described below. Moreover, thalassemia is a severe hereditary anemia that is caused by genetic defects of globin genes and is prevalently associated with HIO. Contrastingly, the pathogenic mechanisms of nonhereditary HIO are yet to be fully elucidated. However, several molecular mechanisms underlying HIO in CLDs have been postulated based on clinical and basic research and are herein presented, followed by a summary of the hepatocarcinogenic potential of HIO.

### Hepcidin-Mediated Regulation of Systemic Iron Metabolism

Hepcidin is a central player in iron metabolism in humans and is mainly expressed and secreted from hepatocytes and binds to ferroportin, a cellular iron exporter, which is present in the cellular membrane of all types of cells involved in systemic iron metabolism, including hepatocytes, macrophages, and enterocytes (7, 8). Upon binding to hepcidin, ferroportin is taken up by endocytosis and degraded in lysosomes (7, 8).

The primary physiological function of hepcidin is to decrease circulating iron levels by inhibiting cellular iron efflux. Dietary iron absorbed by enterocytes is released to the circulation *via* ferroportin and stored mainly in the liver, skeletal muscle, and reticuloendothelial cells. Whereas, aged or injured red blood cells were phagocytosed by liver Kupffer cells and spleen red pulp macrophages (9). Moreover, hepatocytes and Kupffer cells take up hemoglobin released from hemolytic red blood cells (9). The intracellularly stored iron is exported to the circulation *via* ferroportin and utilized for erythropoiesis. Thus, hepcidin obstructs dietary iron absorption, while it also suppresses the release of stored iron, leading to cellular iron accumulation. Thus, hepcidin decreases body iron storage and systemic iron mobilization, and, in some cases, causes iron-deficiency anemia (10).

Hepcidin expression in the liver is tightly regulated by several factors. HFE is a membrane protein that binds to TFR1, competing with holo-transferrin (11). Increased transferrin saturation facilitates the dissociation of HFE from TFR1 and binding of HFE to TFR2, resulting in transferrin-induced hepcidin upregulation (11, 12). Hemojuvelin is a BMP co-receptor required for BMP6-induced hepcidin expression (13, 14). Iron overload upregulates BMP6 in the liver, thereby inducing iron-dependent hepcidin expression *via* the BMP6/hemojuvelin/SMAD pathway (14, 15). It is demonstrated that HFE also binds to BMP type I receptor ALK3 and induces hepcidin expression *via* the SMAD pathway (16). Thus, it is suggested that the BMP/SMAD pathway is a critical regulator of iron metabolism by regulating hepcidin expression. In addition to iron, hepatic hepcidin expression is also induced by inflammatory stimuli, such as interleukin-6 and lipopolysaccharide (17, 18). The increase in hepcidin expression upon inflammation leads to the development of inflammatory anemia, which is characterized by decreases in serum iron and erythropoiesis, despite of increased cellular iron stores in the reticuloendothelial system (10).

### Molecular Mechanisms

HIO is found in 10–36% of patients with chronic hepatitis C, and the hepatic iron amount is associated with a disease severity and decreased by interferon therapy (19, 20). In healthy individuals, hepatic iron accumulation induces hepcidin expression in hepatocytes *via* the BMP pathway to inhibit dietary iron uptake (13, 21). However, patients with chronic hepatitis C show lower hepcidin expression than patients with hepatitis B and nonviral hepatitis despite HIO (22). Moreover, although hepatic inflammation is evident, chronic hepatitis C virus (HCV) infection was shown to downregulate hepcidin (23). This might be due to the impairment of the BMP6/hemojuvelin pathway by TNFα, which suppresses the transcription of hemojuvelin (24). Contrastingly, hepcidin expression was reported to increase in culture cells and experimental animal models of HCV infection (25, 26). At a molecular level, HCV core protein activates the *HAMP* gene promoter while nonstructural protein 5A suppresses it (25, 27, 28). Thus, hepcidin levels in HCV-infected patients might be altered by infection status (acute/chronic, inflammation status, virus load, infection period, etc.) (29).

Alcohol intake is a trigger of systemic iron overload and concomitantly reduces the risk of iron-deficient anemia (30). Increased serum ferritin and transferrin saturation were observed (31, 32) and approximately half develop HIO in patients with alcoholic liver disease (33). Alcohol was shown to suppress hepcidin transcription in cultured cells and laboratory animals, possibly by inhibiting C/EBPα (34, 35). Likewise, decreased serum hepcidin levels and increased intestinal ferroportin expression were depicted in patients with alcoholic liver disease (36, 37), and intestinal iron absorption was consistently increased two-fold in chronic alcoholics (38). Thus, excessive dietary iron absorption due to the decreased hepcidin expression might occur in patients with alcoholic liver disease as well as chronic hepatitis C.

Like chronic hepatitis C, approximately one-third of patients with NAFLD are associated with HIO (39). Iron metabolism alteration results in hyperferritinemia, which is significantly associated with patients with NAFLD (40). Variants of *HFE*, *TMPRSS6*, *HBB*, and *CP* have been reported as genetic factors associated with HIO in patients with NAFLD (41–44); however, nongenetic factors remain unclear. We and other groups have determined hepatic expression levels of iron metabolism-related genes in patients or rats with NAFLD and found the upregulation of hepcidin (45–47). Moreover, hepatic ferroportin expression was downregulated in NAFLD (45, 47, 48). Based on these observations, dysregulated hepcidin expression might suppress hepatic iron export *via* ferroportin in patients with NAFLD. Interestingly, amelioration of HIO, concomitant with the upregulation of hepatic ferroportin expression, was observed in mice fed with a high-fat diet after a fibroblast growth factor 21 treatment (48).

### Hepatocarcinogenic Risk

Hepatic neoplastic nodules were found in 5 of 8 rats fed with an iron-supplemented diet for 32 months and one of the rats with neoplastic nodule developed a HCC, while only 1 of 9 control rats developed neoplastic nodules (49). This iron challenge significantly exacerbated hepatic oxidative stress and DNA damage (50). Adult males in sub-Saharan Africa are often affected with dietary iron overload from a traditional home-brewed beer fermented in steel drums (51). Several lines of studies suggest that there is an association between HCC and dietary iron overload in black Africans (52–54). Consistently, two retrospective studies demonstrated that HCC prevalence in patients with nonalcoholic steatohepatitis (NASH)- or HCV-related cirrhosis is significantly associated with the presence of HIO (55, 56). In particular, iron deposition in the portal tract was significantly associated with poor survival of patients with HCC after curative resection (57). Whereas, phlebotomy with a low-iron diet effectively reduced the risk of development of HCC in chronic hepatitis C patients (58). Thus, HIO has a hepatocarcinogenic potential and is considered a risk factor for HCC development while interventions targeting iron metabolism, such as iron reduction therapy, are promising for prevention of HCC. However, there remains a need for more robust evidence of the hepatocarcinogenic risk of HIO, for example, through long-term follow-up studies.

Liver fibrosis is known as a major risk factor for HCC development (59). Hyperferritinemia in NAFLD patients with HIO independently predicts the risk of advanced liver fibrosis (60). Consistently, predominant parenchymal iron deposition was associated with advanced fibrosis stages in patients with NAFLD (61). However, a contradictory report demonstrated that nonparenchymal iron deposition in patients with NAFLD was more associated with advanced histological features, including fibrosis and inflammation (39). A recent study of 299 patients with NAFLD with a mean follow-up period of 8.4 years demonstrated that nonparenchymal iron deposition more likely leads to fatal hepatic or cardiac disease development (62). However, this study did not show the association between HIO and HCC, possibly due to the insufficient sample size and follow-up period. The clinical significances of parenchymal and nonparenchymal iron depositions remain elusive; however, liver fibrosis could be a key factor in HIO-induced hepatocarcinogenesis.

## Prevention of Iron-Induced Liver Damage by Nutrients

**Figure 1:**
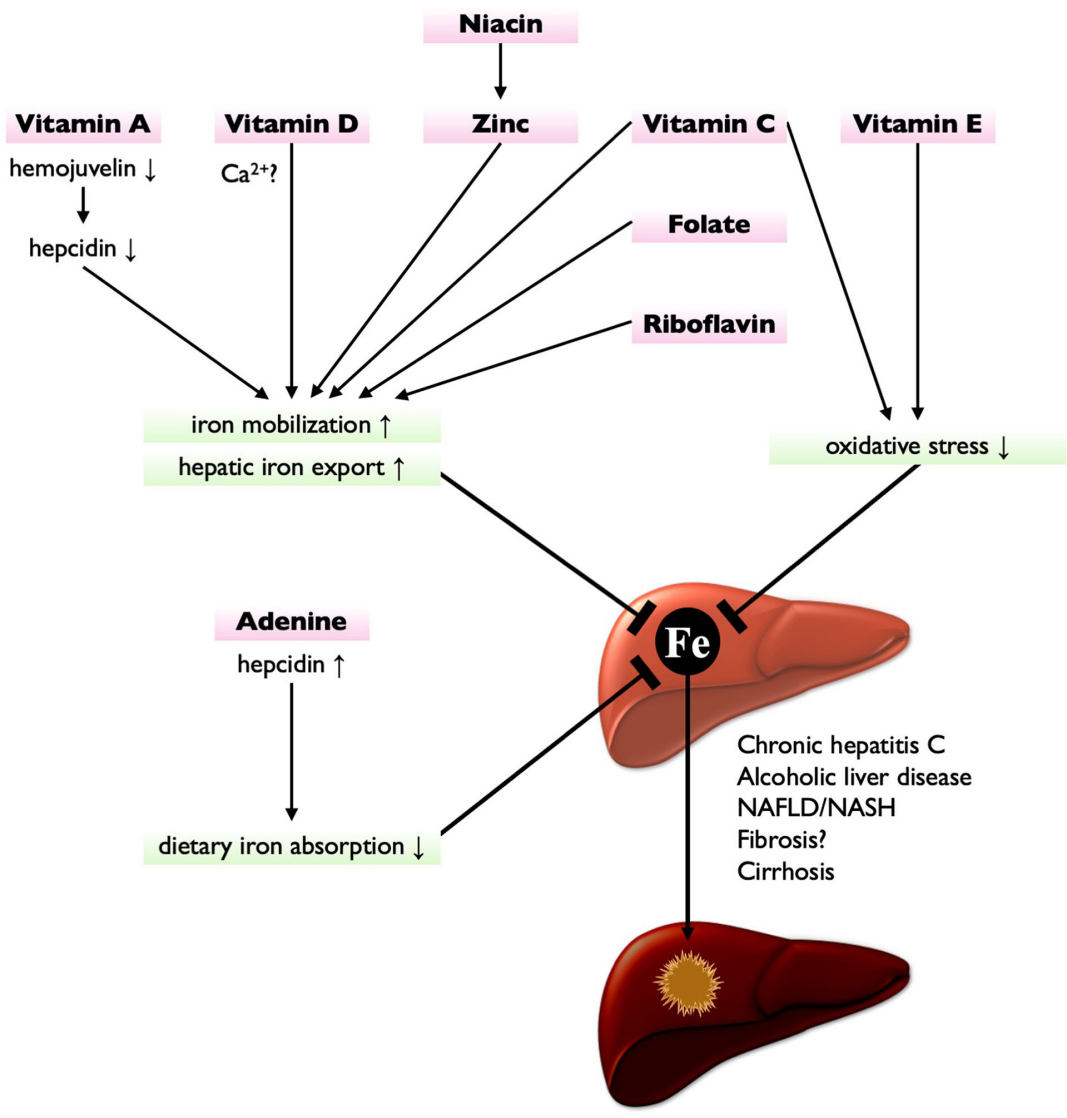
Nutritional interventions targeting iron metabolism for HCC prevention. Vitamin A suppresses hepcidin expression by downregulating hemojuvelin, leading to the enhancement of hepatic iron export. Vitamin D (possibly *via* Ca^2+^ homeostasis), niacin (via zinc), vitamin C, folate, and riboflavin also enhance hepatic iron export and iron mobilization, thereby reducing hepatic iron contents. Vitamin C and vitamin E are potent antioxidant, thereby protecting liver from HIO-induced injury. Adenine increases hepcidin expression, thereby suppressing dietary iron absorption. These nutrients have potential to treat or prevent HIO and may reduce the risk of hepatocarcinogenesis.

HIO would be a therapeutic target to prevent CLD progression. Phlebotomy, indeed, improves disease severity in patients with chronic hepatitis C and reduces the risk of HCC development (58, 63). However, the clinical benefit of phlebotomy has not been established in patients with NAFLD (64). Contrastingly, the dietary iron restriction is shown effective in attenuating liver fibrosis and steatosis in diet-induced NAFLD/NASH model animals (65, 66). Whereas, a negative correlation of hepatic iron contents was observed with dietary intake of vitamins C and E and zinc in patients with thalassemia (67), implying a close relationship between nutritional status and hepatic iron accumulation. Furthermore, several nutrients have been reported to protect the liver from iron-induced damage (**Figure 1**), as discussed below. Therefore, nutritional interventions can be a promising strategy not only for CLD amelioration but also for preventing HIO-induced HCC development.

### Vitamin A

Retinoids are compounds that exert physiological actions of vitamin A. In its active form, retinoic acids, including all-*trans* and other isomers, bind to the retinoic acid receptor and retinoid X receptor and regulate the expression of various target genes. However, we and another group have reported that retinoid signals are metabolically suppressed in NAFLD livers of humans and mice (68, 69). Moreover, retinoid signals are suggested to be epigenetically silenced in HCC by histone lysine-specific demethylase 1 (70). These results suggest that the downregulation of hepatic retinoid signals might be a causative factor for the development and progression of CLDs. Contrastingly, supplementation of all-*trans*-retinoic acid and a synthetic retinoid, tamibarotene, significantly suppressed HIO and hepatic oxidative stress in iron-challenged mice (71, 72). Mechanistically, these retinoids downregulate the hepatic expression of hemojuvelin through retinoic acid receptor α, leading to hepatic hepcidin downregulation and hepatic and intestinal ferroportin upregulation (71, 72). Consequently, hepatic iron export was significantly enhanced (71, 72). Moreover, we observed that the erythroid colony formation unit of bone marrow cells was increased in the presence of all-*trans*-retinoic acid (71). Consistently, retinoids are suggested to have an ability to promote erythropoiesis while their deficiency is associated with anemia (73). Thus, erythropoietic action, at least in part, contributes to HIO amelioration by retinoids. Additionally, retinoids significantly ameliorated hyperglycemia, insulin resistance, and hepatic steatosis in a mouse model of NAFLD (74, 75). Hyperglycemia and hyperinsulinemia, which are frequently complicated with NAFLD, are also risk factors for HCC (76). Thus, from dual aspects, retinoid supplementation would be an efficient strategy to prevent HCC development in patients with NAFLD.

### Vitamin C

Vitamin C is a water-soluble antioxidant required for duodenal cytochrome b to reduce Fe^3+^ to Fe^2+^. This process is necessary for dietary nonheme iron absorption through divalent metal transporter 1 (DMT1) (77). Likewise, vitamin C increased hemoglobin synthesis in patients on hemodialysis with anemia refractory to erythropoietin (78). An observational study with >8,000 Chinese adults showed that dietary vitamin C intake was associated with lower plasma ferritin level (79). These data suggest that vitamin C suppress iron accumulation by enhancing systemic iron mobilization. Contrastingly, in an animal model of alcoholic liver disease, vitamin C supplementation restored the decreased hepcidin expression in the liver and concomitantly downregulated intestinal ferroportin expression, leading to alcohol-induced HIO amelioration (80). Based on these findings, vitamin C is expected to reduce dietary iron absorption in patients with HIO associated with hepcidin downregulation, such as alcoholic liver disease and chronic hepatitis C. However, the nutritional effect of vitamin C on hepatic hepcidin expression and iron mobilization is required further investigation. Moreover, vitamin C was shown to improve glycemic control in patients with type 2 diabetes and NAFLD (81, 82). It is also observed that dietary vitamin C intake was associated with lower HbA1c level (79). Considering its antioxidant effects, vitamin C holds a high potential to prevent HCC development.

### Vitamin D

Vitamin D is a fat-soluble vitamin essential for calcium homeostasis and is produced by ultraviolet light in the dermis or epidermis and activated by successive 25- and 1-hydroxylations in the liver and kidney, respectively (83). 1,25-dihydroxyvitanmin D was shown to protect zebrafish liver cells from ferroptosis, concomitant with decreases in hepcidin expression and cellular iron contents (84). Moreover, it was demonstrated that vitamin D receptor activation inhibits ferroptotic cell death in human renal proximal tubule cells and mouse hippocampal cells (85, 86). However, the protective effects of vitamin D in the liver remains elusive.

Decreased 25-hydroxyvitamin D levels are frequently observed and are associated with diseased severity in patients with CLDs (87–90). The possible explanation of this is that HIO suppresses 25-hydroxyvitamin D production, as its serum levels were negatively correlated with hepatic iron contents in patients with thalassemia major (91–93). Likewise, a negative correlation was found in patients with hereditary hemochromatosis, and 25-hydroxyvitamin D levels were significantly restored after phlebotomy (94). These results suggest that iron is a negative regulator of metabolic activation of vitamin D although its precise mechanism remains unknown. Moreover, vitamin D depletion exacerbated HIO in *hemojuvelin*-knockout mice (95), suggesting that there is a vicious cycle exacerbating HIO by suppressing vitamin D signals. However, 1,25-dihydroxyvitamin D supplementation failed to ameliorate HIO in the *hemojuvelin*-knockout mice (95). Contrastingly, verapamil, a calcium channel blocker, treatment significantly decreased hepatic iron contents and ameliorated HIO-induced liver fibrosis (95, 96). These results suggest that the physiological link between iron and calcium may exist, and that blockade of cellular calcium influx would be relevant for treating HIO. The transport systems of these ions are totally different; however, the involvement of DMT1 is suggested (96, 97). Moreover, duodenum calcium absorption is inversely correlated with duodenum iron absorption and is activated by hepcidin and vitamin D (98). The therapeutic effects of vitamin D supplementation on CLDs are still under debate; however, it is recently suggested that impaired calcium signaling plays a critical role in the development of NAFLD (90, 99). Vitamin D and calcium homeostasis would provide new insights into the pathogenic mechanisms of HIO in CLDs.

### Vitamin E

Tocopherol is a lipophilic antioxidant known as vitamin E, which has been reported to ameliorate steatosis, inflammation, ballooning, and fibrosis in patients with NASH (100, 101). Thus, tocopherol is clinically used for the treatment of NASH. Although its clinical effects on HIO has not been investigated, α-tocopherol significantly reduced hepatic oxidative stress in rats with HIO (102). As its safety and efficacy have been established, it should be investigated whether α-tocopherol also provides clinical benefit for the treatment of HIO in patients with NASH. However, α-tocopherol did not decrease hepatic iron contents in rats with diabetes or with iron overload (102, 103) while it suppressed lipid peroxidation and ferroptosis induced by hepatic ischemia-reperfusion in rats (104). Therefore, the hepatoprotective effects of tocopherol are likely attributable solely to its antioxidant properties.

It was revealed that HIO downregulates miR-122 while upregulating its target gene, CCL-2, leading to hepatic inflammation in iron-challenged rats (102). In contrast to patients with NASH (100), α-tocopherol did not improve inflammation in the iron-challenged rats, possibly because miR-122 and CCL-2 expressions were not restored by α-tocopherol in those rats (102). Thus, α-tocopherol might have a species-specific therapeutic efficacy, suggesting the importance of clinical studies in patients with HIO. Moreover, the suppression of ferroptosis by tocopherol remained to be clarified in patients with CLDs.

### Adenine

Zhang et al. identified adenine as a potent hepatic hepcidin expression inducer from a commercially available vitamin library and found that adenine regulates hepcidin expression *via* the protein kinase A/SMAD pathway (105). Interestingly, adenine significantly ameliorated blood iron parameters and suppressed HIO in mice fed with an iron-enriched diet and *Hfe*-knockout mice, in which hepcidin expression is suppressed (105). Because adenine is clinically used for the treatment of leukopenia, its clinical application for HIO treatment is expected. However, dietary adenine supplementation rapidly induces experimental chronic kidney disease in rodents (106, 107). These animals also develop anemia although serum erythropoietin, which is produced in the kidney, was not altered (107). Whereas, hepcidin was upregulated concomitant with increased serum ferritin and decreased serum iron levels (107). These findings are in agreement with clinical characteristics of inflammatory anemia induced by hepcidin, suggesting that adenine supplementation inhibits iron mobilization by the upregulation of hepcidin. Therefore, the clinical application of adenine for HIO treatment requires optimal dosage determination.

### Zinc

Zinc is a hepatoprotective micronutrient, and its deficiency is suggested to be involved in CLD development and eventually HCC (108). Rats fed with a zinc-deficient diet for 7 weeks developed HIO associated with an increased plasma ferritin level, while zinc intervention returned hepatic iron contents to the normal level (109). The zinc-deficient diet increased plasma hepcidin level, consistent with reduced intestinal iron absorption (109). There might be a physiological crosstalk between iron and zinc in erythropoiesis because clinical studies revealed that patients with iron deficiency anemia were significantly associated with zinc deficiency (110, 111). Moreover, zinc supplementation stimulates erythropoiesis while zinc plus iron more efficiently ameliorated anemia than iron alone (112, 113). These results suggest that zinc ameliorates HIO by enhancing iron mobilization and utilization. The therapeutic effects of zinc on CLDs have been established (108); however, the benefit of zinc supplementation for HIO in patients with CLDs remained unclear.

### Niacin

Dietary nicotinic acid intake was shown to increase intestinal zinc uptake, hepatic zinc and iron contents, and blood hemoglobin levels in weanling rats, thereby promoting their growth (114). Nicotinic acid supplementation restored hepatic zinc to the normal level in rats fed with a low-zinc diet, while depletion of nicotinic acid the low-zinc diet significantly lowered hepatic zinc level (115). These results suggest that nicotinic acid promotes zinc bioavailability; thus, it may ameliorate HIO in patients with CLDs *via* zinc. This point has not been investigated so far. However, nicotinic acid suppresses lipid peroxidation and protects the liver from oxidative stress (115). Thus, nicotinic acid would provide some benefit for patients with CLDs.

Koppe, et al. found that hepatic nicotinamide levels were significantly increased by a dietary iron challenge in mice (116). This was possibly due to iron-induced downregulation of nicotinamide *N*-methyltransferase (NNMT) in hepatocytes (116). Additionally, hepatic NNMT expression was negatively correlated with serum iron parameters in obese individuals (116). Interestingly, NNMT knockdown exacerbated iron-induced damages while its overexpression protected hepatocytes from iron overload (116). NNMT stabilizes NAD^+^-dependent deacetylase SIRT1 by producing *N*^1^-methylnicotinamide *(* 117). SIRT1 regulates various metabolic pathways, and its overexpression ameliorates perturbations of glucose, lipid, and cholesterol metabolisms (117). Thus, *N*^1^-methylnicotinamide is expected as a new nutritional intervention for the treatment of HIO and metabolic syndrome. However, *N*^1^-methylnicotinamide is rapidly inactivated in the liver by aldehyde oxidase. It is demonstrated that the combination of *N*^1^-methylnicotinamide and an aldehyde oxidase inhibitor, hydralazine, significantly ameliorated liver steatosis while *N*^1^-methylnicotinamide alone failed to decrease hepatic triglyceride contents (118).

Clinical research of niacin as a therapeutic for NAFLD is ongoing (119); however, whether niacin ameliorates HIO in patients with CLDs and prevents HCC needs to be addressed by future studies.

### Folate

A recent finding provided a new clue for hepatic heme uptake. Solute carrier family 46 member 1 (SLC46A1) has been suggested to mediate intestinal heme and folate uptake (120). Li et al. investigated its physiological roles by liver-specific SLC46A1 knockdown because its expression is also abundant in the liver (121). In that study, SLC46A1 was shown to uptake heme also in the liver and contribute to the development of HIO in an experimental setting (121). Because SLC46A1 expression was negatively regulated by iron (120, 121), intestinal and hepatic SLC46A1 expression is worth to be determined in patients with CLDs. Interestingly, heme inhibited folate uptake by downregulating SLC46A1 expression while folate did not affect heme uptake and SLC46A1 expression (121), suggesting that folate deficiency is caused by secondary hepatic heme uptake excess. Although folate supplementation unlikely suppressed heme-induced HIO, it promotes iron utilization and mobilization for erythropoiesis. Indeed, it was demonstrated that tissue iron contents including liver and spleen in female rats were significantly lowered by a combined administration of iron and folate, compared with administration of iron alone (122). Therefore, folate supplementation is expected to prevent HIO because of its hematopoietic action.

### Riboflavin

Riboflavin deficiency was shown to reduce intestinal iron absorption and utilization, leading to anemia in humans and rats (123, 124). Consequently, hepatic iron contents were significantly reduced by riboflavin deficiency. Thus, riboflavin antagonists, such as galactoflavin (124), would be expected as a novel therapeutic agent for HIO, unlike other nutrients whose agonistic actions are desired therapeutically. However, there are contradictory studies on the effect of riboflavin on anemia (125–127). Despite that, these findings suggest that riboflavin is a confounder of the systemic iron mobilization. The physiological effects of riboflavin on iron metabolism and its mechanism remain as unanswered questions.

## Conclusions

Considering its potent pro-oxidant nature, dysmetabolism of iron has been suggested as a risk factor for CLD development and progression. Moreover, accumulating evidence indicates that iron also has intrinsic functions that exacerbate CLDs, for example, HCV replication/translation promotion and macrophage activation in NAFLD (29, 128–130). Therefore, iron metabolism is an ideal target for CLD treatment, and eventually, HCC prevention. Nutritional interventions are, in general, considered to provide several benefits for patients, including not only therapeutic effects, but also cost-effectiveness. Therapies targeting iron metabolism with nutrients are expected as an alternative approach to prevent the development of HCC.

## Perspectives

This study has the following limitations: 1. Although several nutrients that are beneficial for HIO amelioration were introduced, there remain many other nutrients that are potentially useful for iron metabolism correction. 2. Clinical efficacies of most nutrients are yet to be clarified, in part because the preventive effects of nutrients on hepatocarcinogenesis require long-term follow-up to confirm them. 3. The concerns that side effects such as iron deficiency and anemia could be caused by nutrients were not sufficiently considered. 4. The nutritional effects were discussed in the same way for all CLD patients even though their HIO could arise from different mechanisms.

The clarification of molecular mechanisms underlying HIO development is quite necessary for each etiology of CLD. Whereas, ferroptosis is currently attracting much attention because of its involvement in the development and progression of many diseases including CLDs and HCC (4). However, research focusing on hepatic ferroptosis has not been undertaken for most of nutrients. Moreover, most nutrients have been studied in their sole use; however, combination of nutrients would show synergistic or additive effects on HIO. These points should be investigated in future studies.

As mentioned above, most nutrients still need robust evidence because of the limited number of clinical and biochemical research. Particularly, their HIO amelioration mechanism needs to be studied to provide a scientific rationale for clinical studies. On this point, hepcidin is an ideal target because of its central role in iron metabolism. However, hepcidin has dual aspects on HIO, namely, it suppresses dietary iron absorption while inhibiting systemic iron mobilization. Therefore, hepcidin upregulation could be useful for HIO prevention while its downregulation could ameliorate or treat HIO. Taking this point into consideration, future studies should be undertaken.

## Author Contributions

The author conceived the review, wrote and reviewed the manuscript, and approved it for submission.

## Funding

This work was supported by Takeda Science Foundation.

## Conflict of Interest

The author declares that the research was conducted in the absence of any commercial or financial relationships that could be construed as a potential conflict of interest.

## Publisher’s Note

All claims expressed in this article are solely those of the authors and do not necessarily represent those of their affiliated organizations, or those of the publisher, the editors and the reviewers. Any product that may be evaluated in this article, or claim that may be made by its manufacturer, is not guaranteed or endorsed by the publisher.

## References

[1] Chen Y Fan Z Yang Y Gu C. Iron Metabolism and Its Contribution to Cancer (Review). Int J Oncol (2019) 54(4):1143–54. doi: 10.3892/ijo.2019.4720 30968149

[2] González-Domínguez Á Visiedo-García FM Domínguez-Riscart J González-Domínguez R Mateos RM Lechuga-Sancho AM. Iron Metabolism in Obesity and Metabolic Syndrome. Int J Mol Sci (2020) 21(15):5529. doi: 10.3390/ijms21155529 PMC7432525 32752277

[3] Fenton HJH. LXXIII.—Oxidation of Tartaric Acid in Presence of Iron. J Chem Soc Trans (1984) 65:899–910. doi: 10.1039/CT8946500899

[4] Paganoni R Lechel A Vujic Spasic M. Iron at the Interface of Hepatocellular Carcinoma. Int J Mol Sc (2021) 22(8):4097. doi: 10.3390/ijms22084097 33921027 PMC8071427

[5] Pietrangelo A. Iron and the Liver. Liver Int (2016) 36(Suppl 1):116–23. doi: 10.1111/liv.13020 26725908

[6] Hino K Yanatori I Hara Y Nishina S. Iron and Liver Cancer: An Inseparable Connection. FEBS J (2021). doi: 10.1111/febs.16208 34543507

[7] Nemeth E Tuttle MS Powelson J Vaughn MB Donovan A Ward DM. Hepcidin Regulates Cellular Iron Efflux by Binding to Ferroportin and Inducing its Internalization. Science (2004) 306(5704):2090–3. doi: 10.1126/science 15514116

[8] Nemeth E Ganz T. Hepcidin-Ferroportin Interaction Controls Systemic Iron Homeostasis. Int J Mol Sci (2021) 22(12):6493. doi: 10.3390/ijms22126493 34204327 PMC8235187

[9] Slusarczyk P Mleczko-Sanecka K. The Multiple Facets of Iron Recycling. Genes (Basel) (2021) 12(9):1364. doi: 10.3390/genes12091364 34573346 PMC8469827

[10] Nielsen OH Soendergaard C Vikner ME Weiss G. Rational Management of Iron-Deficiency Anaemia in Inflammatory Bowel Disease. Nutrients (2018) 10(1):82. doi: 10.3390/nu10010082 PMC5793310 29342861

[11] Schmidt PJ Toran PT Giannetti AM Bjorkman PJ Andrews NC. The Transferrin Receptor Modulates Hfe-Dependent Regulation of Hepcidin Expression. Cell Metab (2008) 7(3):205–14. doi: 10.1016/j.cmet.2007.11.016 PMC2292811 18316026

[12] Gao J Chen J Kramer M Tsukamoto H Zhang AS Enns CA. Interaction of the Hereditary Hemochromatosis Protein HFE With Transferrin Receptor 2 is Required for Transferrin-Induced Hepcidin Expression. Cell Metab (2009) 9(3):217–27. doi: 10.1016/j.cmet.2009.01.010 PMC2673483 19254567

[13] Babitt JL Huang FW Wrighting DM Xia Y Sidis Y Samad TA. Bone Morphogenetic Protein Signaling by Hemojuvelin Regulates Hepcidin Expression. Nat Genet (2006) 38(5):531–9. doi: 10.1038/ng1777 16604073

[14] Meynard D Kautz L Darnaud V Canonne-Hergaux F Coppin H Roth MP. Lack of the Bone Morphogenetic Protein BMP6 Induces Massive Iron Overload. Nat Genet (2009) 41(4):478–81. doi: 10.1038/ng.320 19252488

[15] Kautz L Meynard D Monnier A Darnaud V Bouvet R Wang RH. Iron Regulates Phosphorylation of Smad1/5/8 and Gene Expression of Bmp6, Smad7, Id1, and Atoh8 in the Mouse Liver. Blood (2008) 112(4):1503–9. doi: 10.1182/blood-2008-03-143354 18539898

[16] Traeger L Enns CA Krijt J Steinbicker AU. The Hemochromatosis Protein HFE Signals Predominantly *via* the BMP Type I Receptor ALK3 *In Vivo* . Commun Biol (2018) 1:65. doi: 10.1038/s42003-018-0071-1 30271947 PMC6123693

[17] Wrighting DM Andrews NC. Interleukin-6 Induces Hepcidin Expression Through STAT3. Blood (2006) 108(9):3204–9. doi: 10.1182/blood-2006-06-027631 PMC1895528 16835372

[18] Constante M Jiang W Wang D Raymond VA Bilodeau M Santos MM. Distinct Requirements for Hfe in Basal and Induced Hepcidin Levels in Iron Overload and Inflammation. Am J Physiol Gastrointest Liver Physiol (2006) 291(2):G229–37. doi: 10.1152/ajpgi.00092.2006 PMC2891007 16565419

[19] Boucher E Bourienne A Adams P Turlin B Brissot P Deugnier Y. Liver Iron Concentration and Distribution in Chronic Hepatitis C Before and After Interferon Treatment. Gut (1997) 41(1):115–20. doi: 10.1136/gut.41.1.115 PMC1027238 9274482

[20] Metwally MA Zein CO Zein NN. Clinical Significance of Hepatic Iron Deposition and Serum Iron Values in Patients With Chronic Hepatitis C Infection. Am J Gastroenterol (2004) 99(2):286–91. doi: 10.1111/j.1572-0241.2004.04049.x 15046219

[21] Casanovas G Mleczko-Sanecka K Altamura S Hentze MW Muckenthaler MU. Bone Morphogenetic Protein (BMP)-Responsive Elements Located in the Proximal and Distal Hepcidin Promoter are Critical for Its Response to HJV/BMP/SMAD. J Mol Med (Berl) (2009) 87(5):471–80. doi: 10.1007/s00109-009-0447-2 19229506

[22] Fujita N Sugimoto R Takeo M Urawa N Mifuji R Tanaka H. Hepcidin Expression in the Liver: Relatively Low Level in Patients With Chronic Hepatitis C. Mol Med (2007) 13(1-2):97–104. doi: 10.2119/2006-00057.Fujita 17515961 PMC1869620

[23] Eddowes LA Al-Hourani K Ramamurthy N Frankish J Baddock HT Sandor C. Antiviral Activity of Bone Morphogenetic Proteins and Activins. Nat Microbiol (2019) 4(2):339–51. doi: 10.1038/s41564-018-0301-9 PMC6590058 30510168

[24] Salama MF Bayele HK Srai SS. Tumour Necrosis Factor Alpha Downregulates Human Hemojuvelin Expression *via* a Novel Response Element Within Its Promoter. J BioMed Sci (2012) 19(1):83. doi: 10.1186/1423-0127-19-83 22998440 PMC3500654

[25] Moriya K Miyoshi H Shinzawa S Tsutsumi T Fujie H Goto K. Hepatitis C Virus Core Protein Compromises Iron-Induced Activation of Antioxidants in Mice and HepG2 Cells. J Med Virol (2010) 82(5):776–92. doi: 10.1002/jmv.21661 20336713

[26] Miyachi H Kobayashi Y Relja B Fujita N Iwasa M Gabazza EC. Effect of Suppressor of Cytokine Signaling on Hepcidin Production in Hepatitis C Virus Replicon Cells. Hepatol Res (2011) 41(4):364–74. doi: 10.1111/j.1872-034X.2011.00777.x 21348906

[27] Foka P Dimitriadis A Kyratzopoulou E Giannimaras DA Sarno S Simos G. A Complex Signaling Network Involving Protein Kinase CK2 Is Required for Hepatitis C Virus Core Protein-Mediated Modulation of the Iron-Regulatory Hepcidin Gene Expression. Cell Mol Life Sci (2014) 71(21):4243–58. doi: 10.1007/s00018-014-1621-4 PMC11114079 24718935

[28] Dimitriadis A Foka P Kyratzopoulou E Karamichali E Petroulia S Tsitoura P. The Hepatitis C Virus NS5A and Core Proteins Exert Antagonistic Effects on HAMP Gene Expression: The Hidden Interplay With the MTF-1/MRE Pathway. FEBS Open Bio (2021) 11(1):237–50. doi: 10.1002/2211-5463.13048 PMC7780115 33247551

[29] Foka P Dimitriadis A Karamichali E Kyratzopoulou E Giannimaras D Koskinas J. Alterations in the Iron Homeostasis Network: A Driving Force for Macrophage-Mediated Hepatitis C Virus Persistency. Virulence (2016) 7(6):679–90. doi: 10.1080/21505594.2016.1175700 PMC4991317 27058404

[30] Ioannou GN Dominitz JA Weiss NS Heagerty PJ Kowdley KV. The Effect of Alcohol Consumption on the Prevalence of Iron Overload, Iron Deficiency, and Iron Deficiency Anemia. Gastroenterology (2004) 126(5):1293–301. doi: 10.1053/j.gastro.2004.01.020 15131790

[31] Bell H Skinningsrud A Raknerud N Try K. Serum Ferritin and Transferrin Saturation in Patients With Chronic Alcoholic and non-Alcoholic Liver Diseases. J Intern Med (1994) 236(3):315–22. doi: 10.1111/j.1365-2796.1994.tb00802.x 8077889

[32] Ford C Wells FE Rogers JN. Assessment of Iron Status in Association With Excess Alcohol Consumption. Ann Clin Biochem (1995) 32(Pt 6):527–31. doi: 10.1177/000456329503200602 8579283

[33] Mueller S Rausch V. The Role of Iron in Alcohol-Mediated Hepatocarcinogenesis. Adv Exp Med Biol (2015) 815:89–112. doi: 10.1007/978-3-319-09614-8_6 25427903

[34] Harrison-Findik DD Schafer D Klein E Timchenko NA Kulaksiz H Clemens D. Alcohol Metabolism-Mediated Oxidative Stress Down-Regulates Hepcidin Transcription and Leads to Increased Duodenal Iron Transporter Expression. J Biol Chem (2006) 281(32):22974–82. doi: 10.1074/jbc.M602098200 16737972

[35] Bridle K Cheung TK Murphy T Walters M Anderson G Crawford DG. Hepcidin is Down-Regulated in Alcoholic Liver Injury: Implications for the Pathogenesis of Alcoholic Liver Disease. Alcohol Clin Exp Res (2006) 30(1):106–12. doi: 10.1111/j.1530-0277.2006.00002.x 16433737

[36] Ohtake T Saito H Hosoki Y Inoue M Miyoshi S Suzuki Y. Hepcidin is Down-Regulated in Alcohol Loading. Alcohol Clin Exp Res (2007) 31(1 Suppl):S2–8. doi: 10.1111/j.1530-0277.2006.00279.x 17331161

[37] Dostalikova-Cimburova M Balusikova K Kratka K Chmelikova J Hejda V Hnanicek J. Role of Duodenal Iron Transporters and Hepcidin in Patients With Alcoholic Liver Disease. J Cell Mol Med (2014) 18(9):1840–50. doi: 10.1111/jcmm.12310 PMC4196659 24894955

[38] Duane P Raja KB Simpson RJ Peters TJ. Intestinal Iron Absorption in Chronic Alcoholics. Alcohol Alcohol (1992) 27(5):539–44. doi: 10.1093/oxfordjournals.alcalc.a045289 1476557

[39] Nelson JE Wilson L Brunt EM Yeh MM Kleiner DE Unalp-Arida A. Relationship Between the Pattern of Hepatic Iron Deposition and Histological Severity in Nonalcoholic Fatty Liver Disease. Hepatology (2011) 53(2):448–57. doi: 10.1002/hep.24038 PMC3058264 21274866

[40] Du SX Lu LL Geng N Victor DW Chen LZ Wang C. Association of Serum Ferritin With non-Alcoholic Fatty Liver Disease: A Meta-Analysis. Lipids Health Dis (2017) 16(1):228. doi: 10.1186/s12944-017-0613-4 29197393 PMC5712169

[41] Nelson JE Bhattacharya R Lindor KD Chalasani N Raaka S Heathcote EJ. HFE C282Y Mutations Are Associated With Advanced Hepatic Fibrosis in Caucasians With Nonalcoholic Steatohepatitis. Hepatology (2007) 46(3):723–9. doi: 10.1002/hep.21742 17680648

[42] Valenti L Rametta R Dongiovanni P Motta BM Canavesi E Pelusi S. The A736V TMPRSS6 Polymorphism Influences Hepatic Iron Overload in Nonalcoholic Fatty Liver Disease. PLoS One (2012) 7(11):e48804. doi: 10.1371/journal.pone.0048804 23144979 PMC3489825

[43] Valenti L Canavesi E Galmozzi E Dongiovanni P Rametta R Maggioni P. Beta-Globin Mutations are Associated With Parenchymal Siderosis and Fibrosis in Patients With non-Alcoholic Fatty Liver Disease. J Hepatol (2010) 53(5):927–33. doi: 10.1016/j.jhep.2010.05.023 20739079

[44] Corradini E Buzzetti E Dongiovanni P Scarlini S Caleffi A Pelusi S. Ceruloplasmin Gene Variants Are Associated With Hyperferritinemia and Increased Liver Iron in Patients With NAFLD. J Hepatol (2021) 75(3):506–13. doi: 10.1016/j.jhep.2021.03.014 33774058

[45] Aigner E Theurl I Theurl M Lederer D Haufe H Dietze O. Pathways Underlying Iron Accumulation in Human Nonalcoholic Fatty Liver Disease. Am J Clin Nutr (2008) 87(5):1374–83. doi: 10.1093/ajcn/87.5.1374 18469261

[46] Tsuchiya H Ashla AA Hoshikawa Y Matsumi Y Kanki K Enjoji M. Iron State in Association With Retinoid Metabolism in Non-Alcoholic Fatty Liver Disease. Hepatol Res (2010) 40(12):1227–38. doi: 10.1111/j.1872-034X.2010.00719.x 20880062

[47] Wang C Wang X Song G Xing H Yang L Han K. A High-Fructose Diet in Rats Induces Systemic Iron Deficiency and Hepatic Iron Overload by an Inflammation Mechanism. J Food Biochem (2021) 45(1):e13578. doi: 10.1111/jfbc.13578 33289147

[48] Kim HY Kwon WY Park JB Lee MH Oh YJ Suh S. Hepatic STAMP2 Mediates Recombinant FGF21-Induced Improvement of Hepatic Iron Overload in Nonalcoholic Fatty Liver Disease. FASEB J (2020) 34(9):12354–66. doi: 10.1096/fj.202000790R 32721044

[49] Asare GA Paterson AC Kew MC Khan S Mossanda KS. Iron-Free Neoplastic Nodules and Hepatocellular Carcinoma Without Cirrhosis in Wistar Rats Fed a Diet High in Iron. J Pathol (2006) 208(1):82–90. doi: 10.1002/path.1875 16278820

[50] Asare GA Mossanda KS Kew MC Paterson AC Kahler-Venter CP Siziba K. Hepatocellular Carcinoma Caused by Iron Overload: A Possible Mechanism of Direct Hepatocarcinogenicity. Toxicology (2006) 219(1-3):41–52. doi: 10.1016/j.tox.2005.11.006 16337327

[51] Bothwell TH Seftel H Jacobs P Torrance JD Baumslag N. Iron Overload in Bantu Subjects; Studies on the Availability of Iron in Bantu Beer. Am J Clin Nutr (1964) 14:47–51. doi: 10.1093/ajcn/14.1.47 14106870

[52] Gordeuk VR McLaren CE MacPhail AP Deichsel G Bothwell TH. Associations of Iron Overload in Africa With Hepatocellular Carcinoma and Tuberculosis: Strachan's 1929 Thesis Revisited. Blood (1996) 87(8):3470–6. doi: 10.1182/blood.V87.8.3470.bloodjournal8783470 8605366

[53] Moyo VM Makunike R Gangaidzo IT Gordeuk VR McLaren CE Khumalo H. African Iron Overload and Hepatocellular Carcinoma (HA-7-0-080). Eur J Haematol (1998) 60(1):28–34. doi: 10.1111/j.1600-0609.1998.tb00993.x 9451425

[54] Mandishona E MacPhail AP Gordeuk VR Kedda MA Paterson AC Rouault TA. Dietary Iron Overload as a Risk Factor for Hepatocellular Carcinoma in Black Africans. Hepatology (1998) 27(6):1563–6. doi: 10.1002/hep.510270614 9620327

[55] Chapoutot C Esslimani M Joomaye Z Ramos J Perney P Laurent C. Liver Iron Excess in Patients With Hepatocellular Carcinoma Developed on Viral C Cirrhosis. Gut (2000) 46(5):711–4. doi: 10.1136/gut.46.5.711 PMC1727940 10764717

[56] Sorrentino P D'Angelo S Ferbo U Micheli P Bracigliano A Vecchione R. Liver Iron Excess in Patients With Hepatocellular Carcinoma Developed on non-Alcoholic Steato-Hepatitis. J Hepatol (2009) 50(2):351–7. doi: 10.1016/j.jhep.2008.09.011 19070395

[57] Chung JW Shin E Kim H Han HS Cho JY Choi YR. Hepatic Iron Overload in the Portal Tract Predicts Poor Survival in Hepatocellular Carcinoma After Curative Resection. Liver Int (2018) 38(5):903–14. doi: 10.1111/liv.13619 29105340

[58] Franchini M Targher G Capra F Montagnana M Lippi G. The Effect of Iron Depletion on Chronic Hepatitis C Virus Infection. Hepatol Int (2008) 2(3):335–40. doi: 10.1007/s12072-008-9076-z PMC2716881 19669262

[59] Yip TC Lee HW Chan WK Wong GL Wong VW. Asian Perspective on NAFLD-Associated HCC. J Hepatol (2022) 76(3):726–34. doi: 10.1016/j.jhep.2021.09.024 34619251

[60] Kowdley KV Belt P Wilson LA Yeh MM Neuschwander-Tetri BA Chalasani N. Serum Ferritin Is an Independent Predictor of Histologic Severity and Advanced Fibrosis in Patients With Nonalcoholic Fatty Liver Disease. Hepatology (2012) 55(1):77–85. doi: 10.1002/hep.24706 21953442 PMC3245347

[61] Valenti L Fracanzani AL Bugianesi E Dongiovanni P Galmozzi E Vanni E. HFE Genotype, Parenchymal Iron Accumulation, and Liver Fibrosis in Patients With Nonalcoholic Fatty Liver Disease. Gastroenterology (2010) 138(3):905–12. doi: 10.1053/j.gastro.2009.11.013 19931264

[62] Eder SK Feldman A Strebinger G Kemnitz J Zandanell S Niederseer D. Mesenchymal Iron Deposition Is Associated With Adverse Long-Term Outcome in non-Alcoholic Fatty Liver Disease. Liver Int (2020) 40(8):1872–82. doi: 10.1111/liv.14503 PMC7496452 32378295

[63] Kato J Miyanishi K Kobune M Nakamura T Takada K Takimoto R. Long-Term Phlebotomy With Low-Iron Diet Therapy Lowers Risk of Development of Hepatocellular Carcinoma From Chronic Hepatitis C. J Gastroenterol (2007) 42(10):830–6. doi: 10.1007/s00535-007-2095-z 17940836

[64] Murali AR Gupta A Brown K. Systematic Review and Meta-Analysis to Determine the Impact of Iron Depletion in Dysmetabolic Iron Overload Syndrome and Non-Alcoholic Fatty Liver Disease. Hepatol Res (2018) 48(3):E30–41. doi: 10.1111/hepr.12921 28593739

[65] Abe N Tsuchida T Yasuda SI Oka K. Dietary Iron Restriction Leads to a Reduction in Hepatic Fibrosis in a Rat Model of Non-Alcoholic Steatohepatitis. Biol Open (2019) 8(5):bio040519. doi: 10.1242/bio.040519 31097447 PMC6550076

[66] Crawford DHG Ross DGF Jaskowski LA Burke LJ Britton LJ Musgrave N. Iron Depletion Attenuates Steatosis in a Mouse Model of Non-Alcoholic Fatty Liver Disease: Role of Iron-Dependent Pathways. Biochim Biophys Acta Mol Basis Dis (2021) 1867(7):166142. doi: 10.1016/j.bbadis.2021.166142 33839281

[67] Goldberg EK Neogi S Lal A Higa A Fung E. Nutritional Deficiencies Are Common in Patients With Transfusion-Dependent Thalassemia and Associated With Iron Overload. J Food Nutr Res (Newark) (2018) 6(10):674–81. doi: 10.12691/jfnr-6-10-9 PMC6296481 30569002

[68] Ashla AA Hoshikawa Y Tsuchiya H Hashiguchi K Enjoji M Nakamuta M. Genetic Analysis of Expression Profile Involved in Retinoid Metabolism in Non-Alcoholic Fatty Liver Disease. Hepatol Res (2010) 40(6):594–604. doi: 10.1111/j.1872-034X.2010.00646.x 20618457

[69] Saeed A Bartuzi P Heegsma J Dekker D Kloosterhuis N de Bruin A. Impaired Hepatic Vitamin A Metabolism in NAFLD Mice Leading to Vitamin A Accumulation in Hepatocytes. Cell Mol Gastroenterol Hepatol (2021) 11(1):309–25.e3. doi: 10.1016/j.jcmgh.2020.07.006 32698042 PMC7768561

[70] Kim S Bolatkan A Kaneko S Ikawa N Asada K Komatsu M. Deregulation of the Histone Lysine-Specific Demethylase 1 Is Involved in Human Hepatocellular Carcinoma. Biomolecules (2019) 9(12):810. doi: 10.3390/biom9120810 PMC6995592 31805626

[71] Tsuchiya H Akechi Y Ikeda R Nishio R Sakabe T Terabayashi K. Suppressive Effects of Retinoids on Iron-Induced Oxidative Stress in the Liver. Gastroenterology (2009) 136(1):341–50.e8. doi: 10.1053/j.gastro.2008.09.027 18952085

[72] Yoshikawa O Ebata Y Tsuchiya H Kawahara A Kojima C Ikeda Y. A Retinoic Acid Receptor Agonist Tamibarotene Suppresses Iron Accumulation in the Liver. Obes (Silver Spring) (2013) 21(1):E22–5. doi: 10.1002/oby.20013 23404745

[73] Cañete A Cano E Muñoz-Chápuli R Carmona R. Role of Vitamin A/Retinoic Acid in Regulation of Embryonic and Adult Hematopoiesis. Nutrients (2017) 9(2):159. doi: 10.3390/nu9020159 PMC5331590 28230720

[74] Tsuchiya H Ikeda Y Ebata Y Kojima C Katsuma R Tsuruyama T. Retinoids Ameliorate Insulin Resistance in a Leptin-Dependent Manner in Mice. Hepatology (2012) 56(4):1319–30. doi: 10.1002/hep.25798 22531980

[75] Ebata Y Takino J Tsuchiya H Sakabe T Ikeda Y Hama S. Presence of Glyceraldehyde-Derived Advanced Glycation End-Products in the Liver of Insulin-Resistant Mice. Int J Vitam Nutr Res (2013) 83(2):137–41. doi: 10.1024/0300-9831/a000150 24491887

[76] Plaz Torres MC Jaffe A Perry R Marabotto E Strazzabosco M Giannini EG. Diabetes Medications and Risk of HCC. Hepatology (2022). doi: 10.1002/hep.32439 PMC9790535 35239194

[77] Ganasen M Togashi H Takeda H Asakura H Tosha T Yamashita K. Structural Basis for Promotion of Duodenal Iron Absorption by Enteric Ferric Reductase With Ascorbate. Commun Biol (2018) 1:120. doi: 10.1038/s42003-018-0121-8 30272000 PMC6123691

[78] Attallah N Osman-Malik Y Frinak S Besarab A. Effect of Intravenous Ascorbic Acid in Hemodialysis Patients With EPO-Hyporesponsive Anemia and Hyperferritinemia. Am J Kidney Dis (2006) 47(4):644–54. doi: 10.1053/j.ajkd.2005.12.025 16564942

[79] Luo X Zhang W He Z Yang H Gao J Wu P. Dietary Vitamin C Intake Is Associated With Improved Liver Function and Glucose Metabolism in Chinese Adults. Front Nutr (2022) 8:779912. doi: 10.3389/fnut.2021.779912 35174195 PMC8841761

[80] Guo X Li W Xin Q Ding H Zhang C Chang Y. Vitamin C Protective Role for Alcoholic Liver Disease in Mice Through Regulating Iron Metabolism. Toxicol Ind Health (2011) 27(4):341–8. doi: 10.1177/0748233710387007 21078691

[81] Mason SA Keske MA Wadley GD. Effects of Vitamin C Supplementation on Glycemic Control and Cardiovascular Risk Factors in People With Type 2 Diabetes: A GRADE-Assessed Systematic Review and Meta-Analysis of Randomized Controlled Trials. Diabetes Care (2021) 44(2):618–30. doi: 10.2337/dc20-1893 33472962

[82] He Z Li X Yang H Wu P Wang S Cao D. Effects of Oral Vitamin C Supplementation on Liver Health and Associated Parameters in Patients With Non-Alcoholic Fatty Liver Disease: A Randomized Clinical Trial. Front Nutr (2021) 8:745609. doi: 10.3389/fnut.2021.745609 34595203 PMC8478121

[83] Vitamin D. In: LiverTox: Clinical and Research Information on Drug-Induced Liver Injury. Bethesda (MD: National Institute of Diabetes and Digestive and Kidney Diseases. 31643176

[84] Cheng K Huang Y Wang C. 1,25(OH)2D3 Inhibited Ferroptosis in Zebrafish Liver Cells (ZFL) by Regulating Keap1-Nrf2-GPx4 and NF-κb-Hepcidin Axis. Int J Mol Sci (2021) 22(21):11334. doi: 10.3390/ijms222111334 34768761 PMC8583391

[85] Hu Z Zhang H Yi B Yang S Liu J Hu J. VDR Activation Attenuate Cisplatin Induced AKI by Inhibiting Ferroptosis. Cell Death Dis (2020) 11(1):73. doi: 10.1038/s41419-020-2256-z 31996668 PMC6989512

[86] Li L Li WJ Zheng XR Liu QL Du Q Lai YJ. Eriodictyol Ameliorates Cognitive Dysfunction in APP/PS1 Mice by Inhibiting Ferroptosis *via* Vitamin D Receptor-Mediated Nrf2 Activation. Mol Med (2022) 28(1):11. doi: 10.1186/s10020-022-00442-3 35093024 PMC8800262

[87] Targher G Bertolini L Scala L Cigolini M Zenari L Falezza G. Associations Between Serum 25-Hydroxyvitamin D3 Concentrations and Liver Histology in Patients With Non-Alcoholic Fatty Liver Disease. Nutr Metab Cardiovasc Dis (2007) 17(7):517–24. doi: 10.1016/j.numecd.2006.04.002 16928437

[88] Anty R Canivet CM Patouraux S Ferrari-Panaia P Saint-Paul MC Huet PM. Severe Vitamin D Deficiency May be an Additional Cofactor for the Occurrence of Alcoholic Steatohepatitis. Alcohol Clin Exp Res (2015) 39(6):1027–33. doi: 10.1111/acer.12728 25941109

[89] Gabr SA Alghadir AH. Handgrip Strength and Vitamin D as Predictors of Liver Fibrosis and Malnutrition in Chronic Hepatitis C Patients. Dis Markers (2021) 2021:6665893. doi: 10.1155/2021/6665893 33884041 PMC8041557

[90] Bjelakovic M Nikolova D Bjelakovic G Gluud C. Vitamin D Supplementation for Chronic Liver Diseases in Adults. Cochrane Database Syst Rev (2021) 8(8):CD011564. doi: 10.1002/14651858.CD011564.pub3 34431511 PMC8407054

[91] Wood JC Claster S Carson S Menteer JD Hofstra T Khanna R. Vitamin D Deficiency, Cardiac Iron and Cardiac Function in Thalassaemia Major. Br J Haematol (2008) 141(6):891–4. doi: 10.1111/j.1365-2141.2008.07135.x PMC2892922 18371108

[92] Bajoria R Rekhi E Almusawy M Chatterjee R. Hepatic Hemosiderosis Contributes to Abnormal Vitamin D-PTH Axis in Thalassemia Major. J Pediatr Hematol Oncol (2019) 41(2):e83–9. doi: 10.1097/MPH.0000000000001261 30044347

[93] Yu U Chen L Wang X Zhang X Li Y Wen F. Evaluation of the Vitamin D and Biomedical Statuses of Young Children With β-Thalassemia Major at a Single Center in Southern China. BMC Pediatr (2019) 19(1):375. doi: 10.1186/s12887-019-1744-8 31646984 PMC6813046

[94] Chow LH Frei JV Hodsman AB Valberg LS. Low Serum 25-Hydroxyvitamin D in Hereditary Hemochromatosis: Relation to Iron Status. Gastroenterology (1985) 88(4):865–9. doi: 10.1016/s0016-5085(85)80001-9 3838288

[95] Otto-Duessel M Brewer C Wood JC. Interdependence of Cardiac Iron and Calcium in a Murine Model of Iron Overload. Transl Res (2011) 157(2):92–9. doi: 10.1016/j.trsl.2010.11.002 PMC3073567 21256461

[96] Zhang Y Zhao X Chang Y Zhang Y Chu X Zhang X. Calcium Channel Blockers Ameliorate Iron Overload-Associated Hepatic Fibrosis by Altering Iron Transport and Stellate Cell Apoptosis. Toxicol Appl Pharmacol (2016) 301:50–60. doi: 10.1016/j.taap.2016.04.008 27095094

[97] Chen CC Hsu LW Chen KD Chiu KW Chen CL Huang KT. Emerging Roles of Calcium Signaling in the Development of Non-Alcoholic Fatty Liver Disease. Int J Mol Sci (2021) 23(1):256. doi: 10.3390/ijms23010256 35008682 PMC8745268

[98] Kraidith K Svasti S Teerapornpuntakit J Vadolas J Chaimana R Lapmanee S. Hepcidin and 1,25(OH)2D3 Effectively Restore Ca2+ Transport in β-Thalassemic Mice: Reciprocal Phenomenon of Fe2+ and Ca2+ Absorption. Am J Physiol Endocrinol Metab (2016) 311(1):E214–23. doi: 10.1152/ajpendo.00067.2016 27245334

[99] Ludwiczek S Theurl I Muckenthaler MU Jakab M Mair SM Theurl M. Ca2+ Channel Blockers Reverse Iron Overload by a New Mechanism *via* Divalent Metal Transporter-1. Nat Med (2007) 13(4):448–54. doi: 10.1038/nm1542 17293870

[100] Sanyal AJ Chalasani N Kowdley KV McCullough A Diehl AM Bass NM. Pioglitazone, Vitamin E, or Placebo for Nonalcoholic Steatohepatitis. N Engl J Med (2010) 362(18):1675–85. doi: 10.1056/NEJMoa0907929 PMC2928471 20427778

[101] Gawrieh S Wilson LA Yates KP Cummings OW Vilar-Gomez E Ajmera V. Relationship of ELF and PIIINP With Liver Histology and Response to Vitamin E or Pioglitazone in the PIVENS Trial. Hepatol Commun (2021) 5(5):786–97. doi: 10.1002/hep4.1680 PMC8122381 34027269

[102] Tang Y Jia W Niu X Wu L Shen H Wang L. CCL2 is Upregulated by Decreased miR-122 Expression in Iron-Overload-Induced Hepatic Inflammation. Cell Physiol Biochem (2017) 44(3):870–83. doi: 10.1159/000485355 29176318

[103] Gu Y Lian X Sun W Gao B Fu Y. Diabetes Mellitus Induces Alterations in Metallothionein Protein Expression and Metal Levels in the Testis and Liver. J Int Med Res (2018) 46(1):185–94. doi: 10.1177/0300060517708923 PMC6011322 28760087

[104] Yamada N Karasawa T Wakiya T Sadatomo A Ito H Kamata R. Iron Overload as a Risk Factor for Hepatic Ischemia-Reperfusion Injury in Liver Transplantation: Potential Role of Ferroptosis. Am J Transpl (2020) 20(6):1606–18. doi: 10.1111/ajt.15773 31909544

[105] Zhang Y Wang X Wu Q Wang H Zhao L Wang X. Adenine Alleviates Iron Overload by cAMP/PKA Mediated Hepatic Hepcidin in Mice. J Cell Physiol (2018) 233(9):7268–78. doi: 10.1002/jcp.26559 PMC7754534 29600572

[106] Jia T Olauson H Lindberg K Amin R Edvardsson K Lindholm B. A Novel Model of Adenine-Induced Tubulointerstitial Nephropathy in Mice. BMC Nephrol (2013) 14:116. doi: 10.1186/1471-2369-14-116 23718816 PMC3682934

[107] Kimura T Kuragano T Yamamoto K Nanami M Hasuike Y Nakanishi T. Deregulated Iron Metabolism in Bone Marrow From Adenine-Induced Mouse Model of Chronic Kidney Disease. Int J Hematol (2019) 109(1):59–69. doi: 10.1007/s12185-018-2531-2 30232784

[108] Himoto T Masaki T. Associations Between Zinc Deficiency and Metabolic Abnormalities in Patients With Chronic Liver Disease. Nutrients (2018) 10(1):88. doi: 10.3390/nu10010088 PMC5793316 29342898

[109] Kondaiah P Palika R Mashurabad P Singh Yaduvanshi P Sharp P Pullakhandam R. Effect of Zinc Depletion/Repletion on Intestinal Iron Absorption and Iron Status in Rats. J Nutr Biochem (2021) 97:108800. doi: 10.1016/j.jnutbio.2021.108800 34118433

[110] Ergul AB Turanoglu C Karakukcu C Karaman S Torun YA. Increased Iron Deficiency and Iron Deficiency Anemia in Children With Zinc Deficiency. Eurasian J Med (2018) 50(1):34–7. doi: 10.5152/eurasianjmed.2017.17237 PMC5843450 29531489

[111] Houghton LA Parnell WR Thomson CD Green TJ Gibson RS. Serum Zinc Is a Major Predictor of Anemia and Mediates the Effect of Selenium on Hemoglobin in School-Aged Children in a Nationally Representative Survey in New Zealand. J Nutr (2016) 146(9):1670–6. doi: 10.3945/jn.116.235127 27466609

[112] Chen YH Feng HL Jeng SS. Zinc Supplementation Stimulates Red Blood Cell Formation in Rats. Int J Mol Sci (2018) 19(9):2824. doi: 10.3390/ijms19092824 PMC6165144 30231592

[113] Chen YH Jeng SS Hsu YC Liao YM Wang YX Cao X. In Anemia Zinc is Recruited From Bone and Plasma to Produce New Red Blood Cells. J Inorg Biochem (2020) 210:111172. doi: 10.1016/j.jinorgbio.2020.111172 32659518

[114] Agte VV Paknikar KM Chiplonkar SA. Effect of Nicotinic Acid on Zinc and Iron Metabolism. Biometals (1997) 10(4):271–6. doi: 10.1023/a:1018368231716 9353874

[115] Tupe RS Tupe SG Agte VV. Dietary Nicotinic Acid Supplementation Improves Hepatic Zinc Uptake and Offers Hepatoprotection Against Oxidative Damage. Br J Nutr (2011) 105(12):1741–9. doi: 10.1017/S0007114510005520 21262064

[116] Koppe T Patchen B Cheng A Bhasin M Vulpe C Schwartz RE. Nicotinamide N-Methyltransferase Expression Decreases in Iron Overload, Exacerbating Toxicity in Mouse Hepatocytes. Hepatol Commun (2017) 1(8):803–15. doi: 10.1002/hep4.1083 PMC5678920 29404495

[117] Hong S Moreno-Navarrete JM Wei X Kikukawa Y Tzameli I Prasad D. Nicotinamide N-Methyltransferase Regulates Hepatic Nutrient Metabolism Through Sirt1 Protein Stabilization. Nat Med (2015) 21(8):887–94. doi: 10.1038/nm.3882 PMC4529375 26168293

[118] Takeuchi K Yokouchi C Goto H Umehara K Yamada H Ishii Y. Alleviation of Fatty Liver in a Rat Model by Enhancing N1-Methylnicotinamide Bioavailability Through Aldehyde Oxidase Inhibition. Biochem Biophys Res Commun (2018) 507(1-4):203–10. doi: 10.1016/j.bbrc.2018 30446221

[119] El-Kady RR Ali AK El Wakeel LM Sabri NA Shawki MA. Nicotinamide Supplementation in Diabetic Nonalcoholic Fatty Liver Disease Patients: Randomized Controlled Trial. Ther Adv Chronic Dis (2022) 13:20406223221077958. doi: 10.1177/20406223221077958 35222903 PMC8874180

[120] Le Blanc S Garrick MD Arredondo M. Heme Carrier Protein 1 Transports Heme and is Involved in Heme-Fe Metabolism. Am J Physiol Cell Physiol (2012) 302:C1780–5. doi: 10.1152/ajpcell.00080.2012 22496243

[121] Li H Wang D Wu H Shen H Lv D Zhang Y. SLC46A1 Contributes to Hepatic Iron Metabolism by Importing Heme in Hepatocytes. Metabolism (2020) 110:154306. doi: 10.1016/j.metabol.2020.154306 32621820

[122] Suliburska J Skrypnik K Chmurzyńska A. Folic Acid Affects Iron Status in Female Rats With Deficiency of These Micronutrients. Biol Trace Elem Res (2020) 195(2):551–8. doi: 10.1007/s12011-019-01888-z PMC7176598 31512172

[123] Chen H Kimura M Itokawa Y. Changes in Iron, Calcium, Magnesium, Copper, and Zinc Levels in Different Tissues of Riboflavin-Deficient Rats. Biol Trace Elem Res (1997) 56(3):311–9. doi: 10.1007/BF02785302 9197927

[124] Lane M Alfrey CP Mengel CE Doherty MA Doherty J. The Rapid Induction of Human Riboflavin Deficiency With Galactoflavin. J Clin Invest (1964) 43(3):357–73. doi: 10.1172/JCI104921 PMC441929 14135487

[125] Powers HJ Bates CJ Prentice AM Lamb WH Jepson M Bowman H. The Relative Effectiveness of Iron and Iron With Riboflavin in Correcting a Microcytic Anaemia in Men and Children in Rural Gambia. Hum Nutr Clin Nutr (1983) 37(6):413–25. 6668226

[126] Righetti AA Koua AY Adiossan LG Glinz D Hurrell RF N'goran EK. Etiology of Anemia Among Infants, School-Aged Children, and Young Non-Pregnant Women in Different Settings of South-Central Cote D'ivoire. Am J Trop Med Hyg (2012) 87(3):425–34. doi: 10.4269/ajtmh.2012.11-0788 PMC3435343 22848097

[127] Shi Z Zhen S Wittert GA Yuan B Zuo H Taylor AW. Inadequate Riboflavin Intake and Anemia Risk in a Chinese Population: Five-Year Follow Up of the Jiangsu Nutrition Study. PLoS One (2014) 9(2):e88862. doi: 10.1371/journal.pone.0088862 24533156 PMC3923059

[128] Cho H Lee HC Jang SK Kim YK. Iron Increases Translation Initiation Directed by Internal Ribosome Entry Site of Hepatitis C Virus. Virus Genes (2008) 37(2):154–60. doi: 10.1007/s11262-008-0250-0 18566883

[129] Theurl I Zoller H Obrist P Datz C Bachmann F Elliott RM. Iron Regulates Hepatitis C Virus Translation *via* Stimulation of Expression of Translation Initiation Factor 3. J Infect Dis (2004) 190(4):819–25. doi: 10.1086/422261 15272411

[130] Handa P Thomas S Morgan-Stevenson V Maliken BD Gochanour E Boukhar S. Iron Alters Macrophage Polarization Status and Leads to Steatohepatitis and Fibrogenesis. J Leukoc Biol (2019) 105(5):1015–26. doi: 10.1002/JLB.3A0318-108R 30835899

